# The Experience of Do-Not-Resuscitate Orders and End-of-Life Care Discussions among Physicians

**DOI:** 10.3390/ijerph17186869

**Published:** 2020-09-20

**Authors:** Sheng-Yu Fan, Jyh-Gang Hsieh

**Affiliations:** 1 Institute of Gerontology, College of Medicine, National Cheng Kung University, Tainan 701, Taiwan; 2 Department of Medical Humanities, Tzu Chi University, Hualien 970, Taiwan; jyhgang@gmail.com; 3 Heart Lotus Hospice, Hualien Tzu Chi Hospital, Hualien 970, Taiwan

**Keywords:** cardiopulmonary resuscitation, do not resuscitate, end-of-life care, physicians, patient–doctor communication

## Abstract

Physicians have a responsibility to discuss do-not-resuscitate (DNR) decisions and end-of-life (EOL) care with patients and family members. The aim of this study was to explore the DNR and EOL care discussion experience among physicians in Taiwan. A qualitative study was conducted with 16 physicians recruited from the departments of hospice care, surgery, internal medicine, emergency, and the intensive care unit. The interview guidelines included their DNR experience and process and EOL care discussions, as well as their concerns, difficulties, or worries in discussions. Thematic analysis was used to analyze data. Four themes were identified. First, family members had multiple roles in the decision process. Second, the characteristics of the units, including time urgency and relationships with patients and family members, influenced physicians’ work. Third, the process included preparation, exploration, information delivery, barrier solution, and execution. Fourth, physicians shared reflections on their ability and the conflicts between law, medical professionals, and the best interests of patients. Physicians must consider not only patients’ but also family members’ opinions and surmount several barriers in decision-making. They also experienced negative and positive impacts from these discussions.

## 1. Introduction

In Taiwan, the Hospice Palliative Care Act [1] and Patient’s Right to Autonomy Act [2] have been implemented since 2000 and 2019, respectively. Under these laws, people have the right to make decisions about do-not-resuscitate (DNR) orders when heathy, and physicians have the obligation to tell patients their prognosis and relevant information. The discussion of DNR and end-of-life (EOL) issues could improve the quality of care without heightening symptoms [3].

### 1.1. Theory of Patient-Centered Communication and DNR Discussion

Theory of patient-centered communication addresses four components. First, patient factors include severity of illness, prior illness experience, emotional distress, value, and family. Second, physician factors include personality, risk aversion, autonomy supportiveness, and patient-centered orientation. Third, relationship factors include duration of relationship, trust, and concordance of beliefs. Fourth, health systems factors include access to care, choice of physicians, environment, and waiting times [4].

The DNR discussion is a complex communication process involving above four factors. Physicians have the responsibility to initiate and guide the conversation [5]. Current problems identified in the literature about DNR discussions included physicians who did not conduct such discussions regularly or provide enough time and information, and the discussions that were too late for patients to participate in decision-making [6]. Moreover, medical jargon may be used to describe cardiopulmonary resuscitation (CPR) as a life-sustaining intervention [7], which may be confusing to patients. Talking about death and dying and DNR orders is difficult for physicians, who experience emotional distress and suffering [8,9]. Only 37% of the internal medicine residents felt very comfortable in DNR discussions [10].

### 1.2. Barriers for Physicians in DNR and EOL Discussions

There were several kinds of barriers for physicians. First, patients and family members often lacked the capacity to understand the medical issues, they focused on cures, and their wishes changed at different times or with different physical conditions [10,11,12]. Family tensions and conflicts made it hard to reach consensus, and physicians had difficulty in managing complex family dynamics [11,12]. Second, system barriers included excessive workloads, inefficient information sharing between different departments, and a lack of legal, ethical, and psychosocial support [10,11,13].

Third, physicians’ personal barriers included unresolved feelings about death and dying, fear of taking away hope, fear of damaging the doctor–patient relationship or harming the patient by raising the topic of death, and lack of ability [10,11,12,13]. Fourth, the discussions involved death, specific things not to do, and defeatism, which was also a barrier [11]. In Chinese culture, taboos on the disclosure of death and EOL issues obstruct patient–physician communication, thereby affecting the quality of care [8]. In clinical situations, physicians asked family members to take participate in family conference, and they could deliver information efficiently and solve the conflicts between family members [14].

Resident physicians may be unprepared to handle EOL decision-making, misinterpret patients’ preferences, and their clinical experience is often at odds with what they were taught in formal curricula [15]. More than 90% stated that they would benefit from a formal communication training course [10]. It was useful to explore physicians’ experiences in discussions as part of the foundation of medical education. Therefore, the aim of this study was to explore the experience of DNR and EOL discussions among physicians in Taiwan.

## 2. Methods

### 2.1. Study Design

A qualitative study was conducted. The research paradigm was interpretivism, which was used to explore the subjective experiences of physicians in discussing DNR and EOL issues. Open-ended interview was used to collect data and an inductive approach was used for analysis.

### 2.2. Participants

The participants were physicians who had discussed DNR-related decisions with patients and family members in a medical center in eastern Taiwan. Physicians without relevant experience and those who were not willing to participate in the study were excluded.

Because physicians had these experiences in a variety of departments, purposive sampling was used. The research team began with physicians in the hospice wards. Next, physicians in the internal and surgery departments were invited because they needed to discuss their cases with patients. Finally, physicians in the emergency room (ER) and intensive care units (ICU) were invited, as critical events frequently occurred there.

### 2.3. Data Collection Procedures

Before beginning the study, ethical approval was obtained from the Institutional Review Board of the medical center (IRB number: IRB101-110). The second author was the attending physician in the medical center, and the potential participants were approached in telephone. The aims, procedures, and their rights were explained to the potential participants face to face by the first author. All of the potential participants were willing to participate in the study and signed an informed consent form. They knew the interview was recorded and their personal information would not be identified. They had right to stop interview anytime and did not receive any financial benefits. Data were collected from March 2016 to June 2017.

Semi-structured interviews were used to collect data. The interview guide was developed based on theory of patient-centered communication, involving patients, physicians, relationships, and healthcare system. The interview guide included: (1) the experiences of discussing DNR orders and EOL care with patients and family members, including good and bad experiences; (2) the communication methods usually used; (3) the concerns, difficulties, or worries in discussions of physicians; and (4) support from other healthcare professionals and training about DNR discussions. The interview guide had been tested in a pilot test (one physician) and been modified. The details of open-ended questions are presented in Table A1.

### 2.4. Data Analysis

All interviews were transcribed verbatim. Thematic analysis was used to analyze data [16]. The researchers read the transcripts, familiarized themselves with the contents, and then generated the initial codes. Two researchers gave initial coding separately and then discussed the differences. Coding was based not only on the surface meaning of words but also the context and the ward the physician came from. For example, the same difficult in different wards might have a different impact. Next, potential themes that could capture important data/meaning/descriptions with respect to the research question were explored. For example, the coding about family members were put together and the meaning behind the coding was explored. The quality of themes was evaluated, for example, as to whether there were enough meaningful codes to support the themes, whether the themes provided a thick description related to the research questions, and whether the themes were coherent with clear boundaries. Finally, the themes were named. The analysis was inductive as the coding and theme development were directed by the content of the data. Data collection was until saturation, so that no new theme or meaning emerged.

The first author is a clinical psychologist and the second author is a physician who had more than 15 years’ experience in hospice care. Two researchers agreed the idea that patients had the right to know the truth of diseases and make decision for themselves. However, the differences in working patterns and difficulties about discussions between different departments were also observed in clinical experiences. After each interview, the researcher finished a field note and personal reflection within 24 h. The researchers constantly moved between the transcripts, codes, and themes in the process of analysis. The researchers also compared the transcripts and their field notes for accuracy. The researchers reviewed the multiple sources of materials and kept reminding them that the analysis had to be based on the participants’ interviews. In addition, five participants were invited to review the results and provide feedback to ensure the accuracy and credibility of the analysis. Atlas.ti 6.0 was used for data analysis.

## 3. Results

Sixteen physicians were recruited with ages ranging from 28 to 42 years, and all participants received interview one time. Nine were attending physicians, and their years as practicing physicians ranged from 3 to 14 years. The details of their demographic and work characteristics are presented in Table 1. Four themes were identified, including (1) multiple roles of family members in discussions, (2) impact of the ward processes on EOL discussions, (3) process of discussion, and (4) professional ability and reflections. The major concerns included family members and the word processes. The participants developed a certain process of discussion form clinical experience, and they also had reflections about their own ability and roles in discussion.

### 3.1. Multiple Roles of Family Members in Discussions

The participants spent a large part of each interview discussing family members, who were a major concern in DNR and EOL care discussions. Family members had multiple roles, including information receivers, collaborative decision-makers, blockers, and the persons to be caring for patients. As information receivers, family members needed the information about the patients’ prognosis and future care plans. As collaborative decision-makers, family members were involved in the discussion and decision-making process. When the patients’ physical condition and consciousness status did not allow them to make decisions, family members had to take responsibility for decision-making.

As blockers, family members impeded physicians’ approaching the patients and did not allow them to discuss EOL matters with patients. Family members who were not primary caregivers might not understand a patients’ physical conditions, future prognosis, or they did not understand the patients’ suffering or painful experiences. They worried about the negative impacts on patients, such as loss of fighting spirit, negative emotions, and refusal of future treatment. Facing a relative’s death was difficult, and they could not accept the fact that patients were near death; they would ask physicians and the team to take any measures that might prolong patients’ lives. As the persons to be caring for patients, some family members had strong emotional responses, such as anger or anxiety, and avoided the discussion. Their emotional response frequently meant denial or an inability to accept patients’ death.

K: “One of the most important considerations is family members’ attitudes. They may not want us to tell the patient. They have a lot of worries, such as losing fighting spirit or having negative feelings. Even when some family members agree with the ideas of DNR and hospice care, they will make decisions when patients are in coma.”

H: “Hospice care addresses ‘care for the whole family,’ and family members who cannot accept patients’ death are our target of care. Their resistance or denial have psychological meaning.”

Meanwhile, family dynamics influenced the discussion and decision. There were different opinions amongst family members about the EOL decisions, and conflicts between family members was one of the barriers that physicians had to address. One physician said (E), “we have to be aware of the family dynamics because most of time there is not just one family member who will make the final decision.”

### 3.2. Impact of the Ward Processes on EOL Discussions

There were different approaches in different wards, including “time urgency” and “familiarity with patients and family members.” Time urgency meant the decision had to be made in a short time. In ER and ICU, physicians needed the decisions immediately to give treatment. One physician said (I): “we do not have time to wait, we cannot give family members much time to think. Just five minutes, five minutes, and then we must take action.”

In contrast, physicians on the hospice palliative care teams may visit patients several times to solve the barriers. A physician on the hospice ward said (H): “I usually start by understanding patients’ symptoms and their main concerns, and explore their opinions on DNR and hospice care. If needed, I can come a second or third time.”

Familiarity with patients and family members meant previous experience with patients and family members, so that physicians knew patients’ treatment process and understood patients and family members’ characteristics. Physicians in the oncology department usually had a long period with patients in which they built relationships of trust. For physicians in the ER and ICU, however, it might be the first time they had seen the patients, and they had to make decisions based on their current information and family members’ opinions.

N: “We have never met the patients before, and the first time is when patients’ physical condition is very bad. After physical examinations and reading the medical chart, we inform family members and ask them to make a decision. We have no idea about the patients or their illness experience.”

In addition, workload was an important consideration. A majority of the participants mentioned that they had to prioritize their work. Most of the time physical care and treatment came first. If they needed to spend a lot of time in DNR discussion, they accepted and followed the first decision that patients and family members had made.

N: “We might not have so much time, and only provide care based on their first decision, whether for DNR or to continue CPR.”

### 3.3. Process of Discussion

There was a process of discussion. First, physicians noted what was needed for the discussion and prepared the relevant physical information and data. Second, they explored patients’ and family members’ willingness to discuss EOL issues and decided an appropriate time. Regarding the timing of discussion, patients often did not consider DNR issues to be relevant or found it hard to think about when their physical condition is good; but they also found it difficult to accept when close to death. Physicians in the oncology or internal medicine departments tended to raise the issue when their physical condition had started to decline, which ensured that patients and family members had time to think about it.

M: “When people are healthy, they may not be aware of the necessity of this issue; however, when their physical condition is very bad, they may not have the ability or time to think and discuss it. I used to open the discussion when the disease had progressed to the advanced stage.”

Third, they delivered a variety of information. The information package included the diagnosis and prognosis, future disease trajectory and life expectancy, the process of dying and symptoms, possible treatment plans, the limitations of medical, hospice, and palliative care, CPR and life-sustaining treatment, DNR and its consequences, the effects and side effects of CPR, and the goals of care and care planning. CPR events included endotracheal intubation, cardiac massage, artificial nutrition, and hydration. The contents of information were based on patients’ and family members’ needs and physical conditions.

Fourth, physicians had to surmount the barriers to discussion. Physicians tried to establish relationships of trust with patients and family members as the foundation of dialogue. In rational terms, they gave precise and definite information such as the probability or rate of survival in dying patients after receiving CPR. However, they had to deal with patients and family members’ emotional responses and explore the real concerns or worries behind the emotions.

O: “It involves not only rational thinking but also emotional feeling. We had to empathize with their feelings and difficulties. This may take time and we need help from psychosocial care professionals.”

Because of the significant roles of family members in EOL decisions, family conferences were frequently used to solve the conflicts between patients and family members. In addition, they needed psychologists and social workers’ help to solve psychosocial and family dynamic issues. It was not easy to accept the nearness of death and make an appropriate decision, and they needed time to think about the issues.

K: “Some family members did not realize patients’ physical conditions, or there were differences of opinion between family members, and then I would call family conferences where I could tell all the family members at the same time and give them a chance to discuss it.”

In the end, physicians in non-hospice wards followed the decision for DNR or continuing CPR. If patients and family members did not accept a DNR order, then they gave CPR at the patient’s death based on the law.

F: “After all, we should follow the law. If they did not accept DNR, then we had the responsibility to give CPR.”

### 3.4. Professional Ability and Reflections

Some of the participants had received communication training courses in school, but most of them stated that they learned communication skills from their clinical experience. They needed to think through the process of discussion, their good or bad points, and ways they could improve. Furthermore, the physicians expressed several reflections. First was the nature of a DNR and the best interests of patients. Physicians should not give futile medical treatments that only caused harm rather than cure to patients close to death. Patients had a right to make decisions for themselves. However, patients might not wish to face decisions related to death. One physician (B) stated: “Sometimes when we told patients that DNR is better for them, they could not accept it. When patients come to the hospital, what they want is to be cured. They did not think about death.”

The second issue was whether family members had the power to stop physicians telling the truth and discussing it with patients, and then they made a decision when patients lost consciousness.

A: “In general, we respect family members’ opinions; however, we also have duty by law to tell patients the truth, and ethically we should respect patients’ autonomy. It is definitely a barrier if family members do not want you to tell them. Do family members have a higher power than patients themselves?”

The third concerned the struggles between the law, medical professionals, and the best interests of patients. Several physicians stated that if patients did not agree to a DNR order, they had to perform CPR. However, as medical professionals, they knew it would not be successful or have any benefit, so they questioned whether they had to perform CPR procedures to the end.

I: “We knew CPR would be harmful and not succeed in saving the patient’s life, so why could we not just stop and let the patient die peacefully?”

Fourth was the balance between telling the truth clearly and keeping hope. Physicians would like to deliver clear information; however, that was difficult to do while still helping patients and family members keep hope, as well as to relieve the impact of negative emotions.

O: “We want patients to continue hoping, not for cure, but for the company, that we will always be with them. However, facing death can cause fear, anxiety, uncertainty, and other negative feelings, which makes telling them difficult.”

## 4. Discussion

This study used a qualitative methodology to explore physicians’ experiences in DNR and EOL discussions. Physicians considered family members to have multiple roles in discussions. Discussion was a dynamic process, which time urgency and relationships with patients and family members influenced their discussion. Physicians self-reflected about their abilities and the meaning of discussions.

The discussion was usually held at a time of transition, and physicians delivered information on the limitations of curative treatment and offered a new direction of treatment [17]. Similar to previous study [18], the results of this study showed that family members were generally involved in EOL discussions and had a lot of impact on decision-making. In a family-oriented culture, family members may ask physicians to tell them the diagnosis or prognosis before the patients, and let the family decide whether to tell patients [19,20]. Sometimes they may avoid telling patients the truth to protect them out of worries that the patient cannot handle the truth and would have an emotional reaction [21]. However, these worries constituted a barrier for physicians [11].

In a familial model of decision-making, physicians not only considered patients’ willingness but had to embed the decision in familial relationships [22]. Some physicians told family members about imminent death and DNR/CPR options only when patients became terminal [23]. Furthermore, physicians might need to inhibit family members’ influence on decision-making [24] or strike a balance between family members’ wishes and the appropriateness of care [9]. Family conferences were often used to improve communication and decision-making between patients and family members [14].

ICU and ER had unique characteristics. Patients usually lack decision-making capacity due to their physical condition; family members bear high stress due to the complicated clinical situation, and physicians often do not have a prior relationship with patients [25]. The specifics of the departments influenced physicians’ behaviors, such as how long they could wait, how many times to visit, whether they needed to get the decisions and make a response quickly, and whether they had a relationship of trust as a foundation to directly open the discussion. The relationships between healthcare professionals, patients, and family members were important as a foundation of discussion [26].

As with clinical guidelines, there was a certain process of discussion [5,27,28]. Physicians had to initiate the discussion when patients and family members seemed ready, explore what they already knew and what they wanted to know, and give them opportunities to discuss the future [26]. Patients needed information on medical procedures, outcome probabilities, and the goals of care to make a decision [29]. Sometimes physicians could not give clear information due to the uncertainty of prognosis [11] and the survival rate of CPR [30]. Just delivering information was insufficient [7]; physicians had to be sensitive to and deal with emotional reactions, emphasizing what they could do [31], as well as try to maintain the sense of hope [8]. In the end, they took action based on the patients’ decisions. On the other hand, a trust relationship was as the foundation of discussion, and previous studies revealed physicians had to establish partnerships with patients and family members for discussing and decision-making [32,33].

Physicians in different departments needed to cooperate. Physicians in hospice wards needed those in oncology to introduce them and open the discussion. However, previous studies showed that conflicts between professionals, such as priority of care, role overlap and blurring of responsibility, and lack of communication, could hinder collaboration [5,9]. In addition, physicians needed teamwork for EOL communication with various kinds of healthcare professionals, such as nurses, psychologists, and social workers, who could be aware of patients’ physical or psychosocial needs and manage the difficulties in discussion [34].

Similar to previous study, physicians had internal conflicts about roles and duties between what they had to do and what was best for patients [24]. They had the sense of avoiding relevant discussions [34] because of the nature of discussion and worry about harm on patients. On the other hand, they had positive growths, for example, learning skills, working as a team, gaining appreciation from patients, and self-growth through the discussion process [9].

Regarding clinical implication, physicians have to develop communication skills and sensitivity [26]. They can learn from experiences to develop their own discussion process. They also need educational trainings about how to establish trust relationships with patients and family members, solving the barriers, delivering information, and dealing with emotional reactions. The opinions of family members are one of the major concerns in discussion, and they can learn to conduct family conference. The hospital policy can provide support from a palliative care team and psychosocial professionals.

Some limitations should be acknowledged. First, all the participants were recruited from one medical center and shared the same policy of discussion. Second, these participants were willing to discuss DNR and EOL issues in their clinical work, and those who tended not to discuss them did not participate in the study. A future study can address the roles of family members in decision-making and the issue of educational training on specific barriers.

## 5. Conclusions

Discussion was a dynamic process, and physicians had to consider the multiple roles of family members, the characteristics of work units, their communication abilities, and the team support needed from psychosocial professionals.

## Figures and Tables

**Table 1:** Demographic and work characteristics of the participants.

No	Age	Sex	Professional Backgrounds and Work Places	Position	Working Years	Cases of DNR Discussion a Month	Time of Interview (Minutes)
A	32	Male	Internal Medicine, Division of Chest & Intensive Care Unit	Resident	5	6–10	35
B	30	Male	Family Medicine & Hospice Ward	Resident	4	more than 10	42
C	30	Male	Internal Medicine, Division of Chest	Resident	3	1–5	46
D	39	Male	Division of Colon and Rectal Surgery	Attending Physician	12	1–5	57
E	42	Male	Division of General Surgery & Intensive Care Unit	Attending Physician	14	1–5	47
F	33	Female	Internal Medicine, Division of Chest & Intensive Care Unit	Attending Physician	9	more than 10	62
G	30	Female	Division of Otorhinolaryngology (ENT)	Resident	5	1–5	56
H	41	Male	Family Medicine & Hospice Ward	Attending Physician	8	more than 10	40
I	37	Male	Emergency Department	Attending Physician	11	more than 10	53
J	30	Male	Emergency Department & Pediatric Intensive Care Unit	Resident	4	1–5	53
K	37	Male	Division of Hematology and Oncology	Attending Physician	13	more than 10	57
L	28	Male	Family Medicine & Hospice Ward	Resident	2	1–5	34
M	38	Male	Division of Hematology and Oncology	Attending Physician	13	6–10	45
N	38	Male	Emergency Department	Attending Physician	13	more than 10	47
O	29	Female	Family Medicine & Hospice Ward	Resident	3	1–5	48
P	36	Male	Internal Medicine, Division of Nephrology	Attending Physician	9	1–5	50

## Appendix A

**Table A1:** The open-ended questions.

The Open-Ended Questions
Would you please describe your experiences of discussing DNR orders and EOL care?
What were the clinical situation, patients’ conditions, and the reasons to discussion?
How did you communicate with patients and family members? The preparation before communication, the information and method about communication, and the decision-making and the execution.
What were the concerns, difficulties, or worries in discussions, about patients, family members, yourself, and different departments?
How did you collaborate with staff in other departments?
What were the supports from other healthcare professionals you needed? How did you learn to discuss DNR orders and EOL care? What the training did you need?
